# Bony Stroke: Clinical Features, Management, and Outcomes in a Case Series of Seven Patients

**DOI:** 10.7759/cureus.103369

**Published:** 2026-02-10

**Authors:** Khadija Ouchen, Naima Chtaou, Sara Zejli, Siham Bouchal, Aouatef El Midaoui, Youssef Alaoui Lamrani, Abdellatif Oudidi, Mustapha Maaroufi, Faouzi Belahsen

**Affiliations:** 1 Neurology, Hassan II University Hospital Centre, Fez, MAR; 2 Laboratory of Epidemiology and Research in Health Sciences, Faculty of Medicine, Pharmacy, and Dentistry of Fez, Sidi Mohamed Ben Abdellah University, Fez, MAR; 3 Radiology, Hassan II University Hospital Centre, Fez, MAR; 4 Ear, nose, and throat, Hassan II University Hospital Centre, Fez, MAR

**Keywords:** bone, eagle syndrome, etiology, stroke, young

## Abstract

Bony stroke is defined as a stroke caused by a conflict between the carotid or vertebral arteries and an osseous or fibrous structure. This conflict can be permanent or triggered by specific head positions. It represents an uncommon cause of stroke in young individuals. Diagnosis requires multimodal imaging with both static and dynamic vascular assessments.

We report a retrospective case series of seven patients in whom a bony or structural anomaly was identified as the etiology of stroke after exclusion of other possible causes. The mean age was 50.5 years. Bony anomalies included vascular Eagle syndrome in five patients, with one case of bilateral internal carotid artery conflict with elongated styloid processes and two cases of carotid compression by the hyoid bone. Curative anticoagulation was prescribed in most patients, and surgical removal of the bony anomaly was performed in four cases. No stroke recurrence was observed in our series. One patient died from septic shock.

This study highlights the importance of investigating structural abnormalities in young patients with stroke, particularly in cases of recurrence in the same arterial territory, since appropriate treatment can eliminate the risk of recurrence.

## Introduction

Stroke is a major public health problem worldwide. It is considered the third leading cause of death and the fourth most common cause of disability worldwide in 2021. Ischemic stroke is the most frequent subtype [1]. Recently, significant progress has been made in the management of ischemic stroke, particularly in the etiological workup, which has contributed to improved outcomes.

The carotid and vertebral arteries supply the brain with blood. Because of their anatomical position in the cervical region, these vessels may be vulnerable to conflict with osseous and fibrous structures. When these mechanical conflicts result in a stroke, they are referred to as bony strokes. This diagnosis should be considered in young patients presenting with cryptogenic ischemic stroke, mainly when recurrences occur within the same vascular territory [2,3]. Appropriate diagnosis and management can reduce, or even eliminate, the risk of recurrence [2].

There are several subtypes of bony stroke: vascular Eagle syndrome, defined as a conflict between an abnormally elongated styloid process and the carotid artery; bow hunter syndrome, characterized by vertebrobasilar insufficiency resulting from dynamic compression of the vertebral artery during head rotation, most commonly caused by an osseous structure, although other primary and secondary etiologies have also been reported; and other subtypes involving the thyroid cartilage, hyoid bone, or occiput. The vascular conflict may be permanent or triggered by certain head positions [2,4].

This study aims to characterize the clinical presentation, imaging findings, and management of bony stroke in patients admitted to our center and to describe the underlying arterial-structural conflicts.

## Materials and methods

This retrospective study was conducted over three years, from March 2022 to March 2025, in the neurology department at the University Hospital of Fez, Morocco. We included all patients admitted for management of ischemic stroke in whom a conflict between an artery and osseous or fibrous structure was identified as the etiology of their stroke after ruling out all possible causes. Patients whose stroke was not clearly attributable to a conflict with an osseous or fibrous structure, including those with an osseous anomaly but without confirmed causality, were excluded.

Demographic characteristics (including age, sex, and comorbidities), medical history, clinical manifestations, neuroimaging, and therapeutic data were extracted from electronic medical records (SIH HOSIX). All patients underwent non-contrast CT, CT angiography (CTA), and a cardiac workup. All CTAs were independently reviewed by the same senior neuroradiologist to confirm the osseous conflict underlying the ischemic stroke and to exclude alternative diagnoses. All patients were monitored in the neurovascular day hospital of our department. Neurological outcomes were assessed using the National Institutes of Health Stroke Scale (NIHSS) and the modified Rankin Scale (mRS) [5,6].

Data were entered and analyzed using Microsoft Excel 2010 (Microsoft Corp., Redmond, WA, USA). An anonymized database was created to ensure that no patient could be identified. Some cases included in this series have been previously published as individual case reports by members of our department; these cases were intentionally included to provide a comprehensive departmental overview of bony stroke [7,8].

## Results

This retrospective case series included seven patients, comprising two females (28%) and five males (72%). The mean age was 50.5 years (range 37-66). Four patients had no significant past medical history and no conventional vascular risk factors, including hypertension, diabetes mellitus, dyslipidemia, or tobacco use. One patient was a chronic smoker, and another had a history of hypertension. One patient in the series had previously experienced an ischemic stroke six years earlier due to an extracranial right internal carotid artery (ICA) dissection of unknown etiology, complicated by post-stroke epilepsy. At the time of the current event, he was receiving aspirin (100 mg/day) and carbamazepine, with a pre-event mRS score of 1. None of the patients had a known systemic or connective tissue disease.

The mean time from symptom onset to admission was 19 hours (range 2-72 hours). Three patients were admitted within nine hours of symptom onset. The NIHSS score at admission ranged from six to 17, with a mean of 12. All patients underwent non-contrast CT and CTA of the supra-aortic trunks and the circle of Willis. A non-contrast CT scan revealed subacute ischemic lesions in the following territories: the middle cerebral artery (MCA) territory in four patients; the anterior cerebral artery (ACA) and ACA-MCA junctional territories; and the ACA and MCA territories in one patient. Three patients had chronic ischemic lesions. Two patients showed no evidence of subacute stroke, with an ASPECTS score of 10.

**Figure 1:**
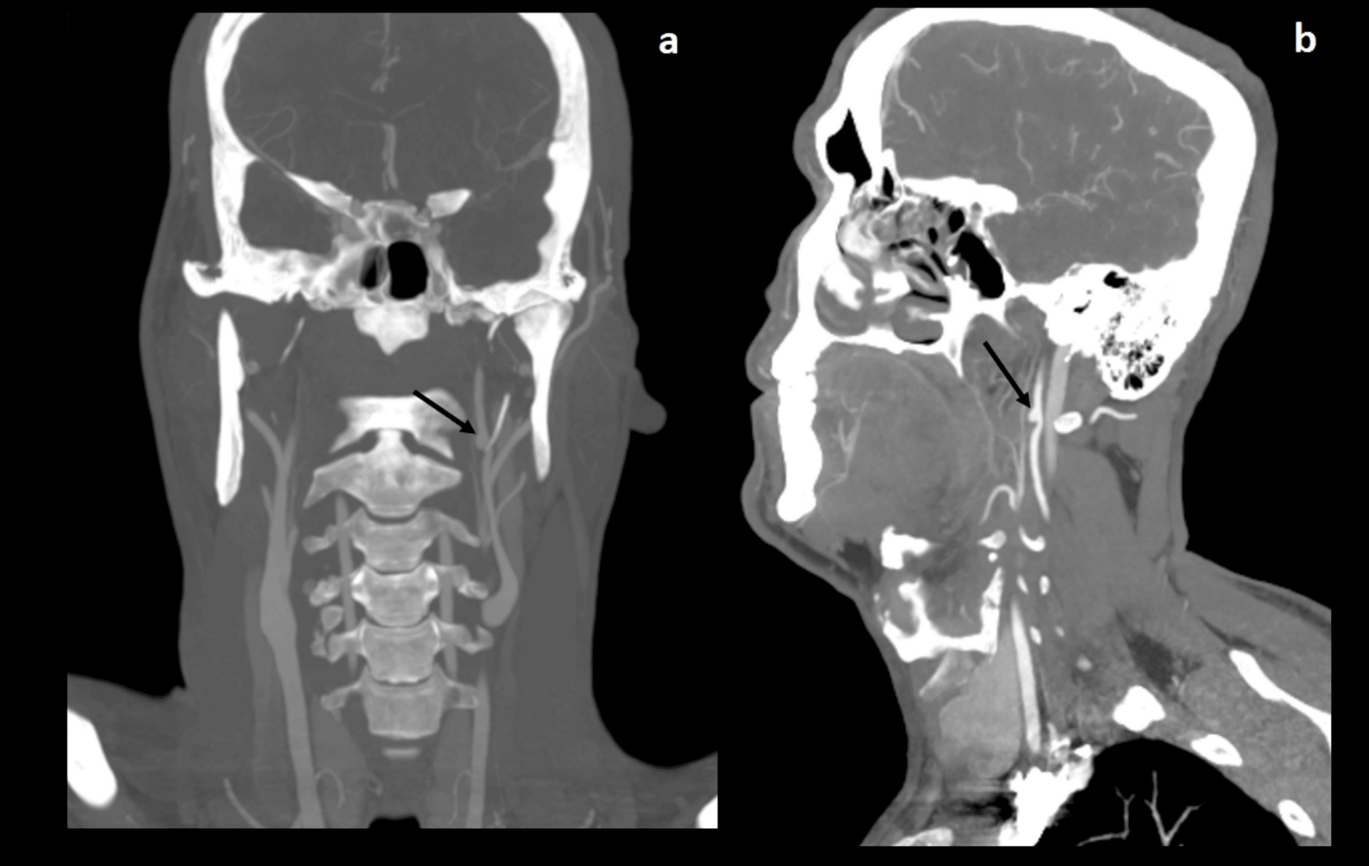
Case 1 coronal (a) and sagittal (b) CT scans demonstrating a pseudoaneurysm of the left ICA (black arrows) CT: computed tomography, ICA: internal carotid artery

**Figure 2:**
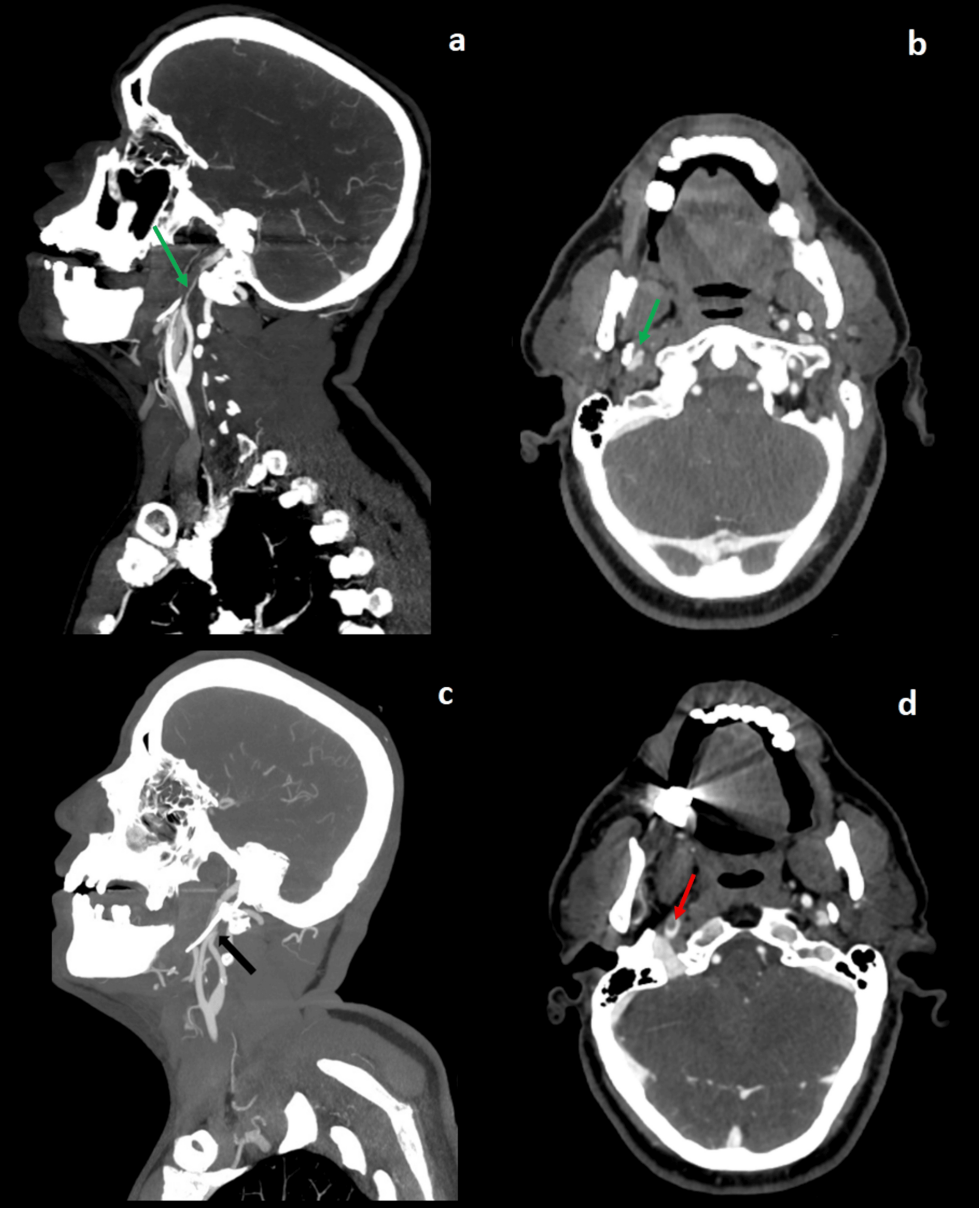
Case 2 sagittal (a, c) and axial (b, d) CT images showing an ICA dissection (green arrows) due to mechanical conflict with an elongated styloid process (c) (black arrow) complicated by a floating thrombus (d) (red arrow) CT: computed tomography, ICA: internal carotid artery

**Figure 3:**
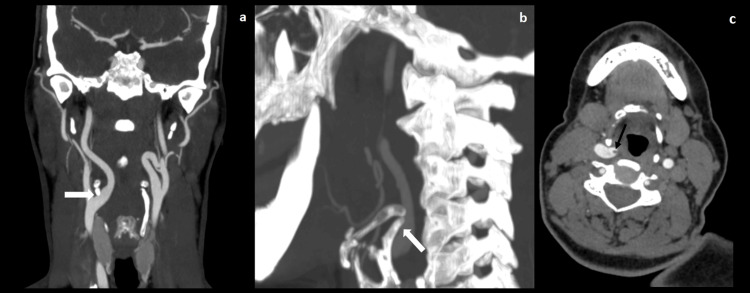
Case 4 frontal (a), sagittal (b), and axial (c) CT scans demonstrating compression of the right ICA by the greater horn of the hyoid bone (white arrows) complicated by a floating thrombus (black arrow) CT: computed tomography, ICA: internal carotid artery

**Figure 4:**
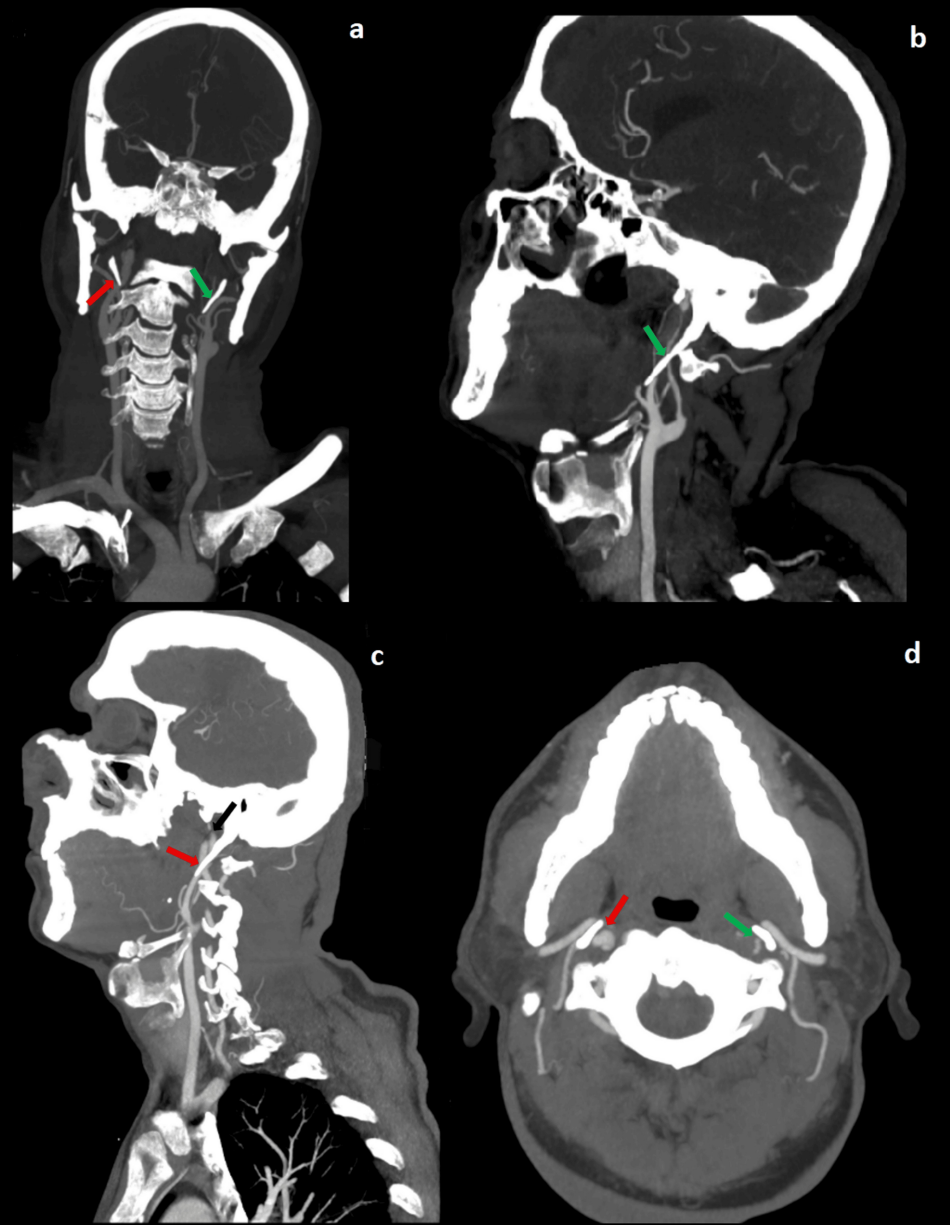
Case 6 frontal (a), sagittal (b c), and axial CT (d) images showing bilateral carotid artery dissection caused by conflict with elongated styloid processes (42.3 mm on the left and 41.3 mm on the right). Red arrows indicate the right carotid artery, and green arrows indicate the left carotid artery. The conflict results in a pseudoaneurysm of the right ICA (black arrow) CT: computed tomography, ICA: internal carotid artery

CTA revealed the following: ICA aneurysm, pseudoaneurysm, or dissection due to a conflict with an elongated styloid process (length >30 mm), consistent with vascular Eagle syndrome, in four patients; a conflict between the ICA and an elongated styloid process, without evidence of aneurysm or dissection, in one patient (confirmed by velocity acceleration in the ICA during positional maneuvers on dynamic ultrasound examination); and ICA floating thrombus or dissection resulting from mechanical conflict with the hyoid bone in two patients. The patient who had an ischemic stroke six years ago was diagnosed with bilateral vascular Eagle syndrome. Figures 1-4 show CT scans demonstrating bony abnormalities observed in patients in our study.

**Figure 5:**
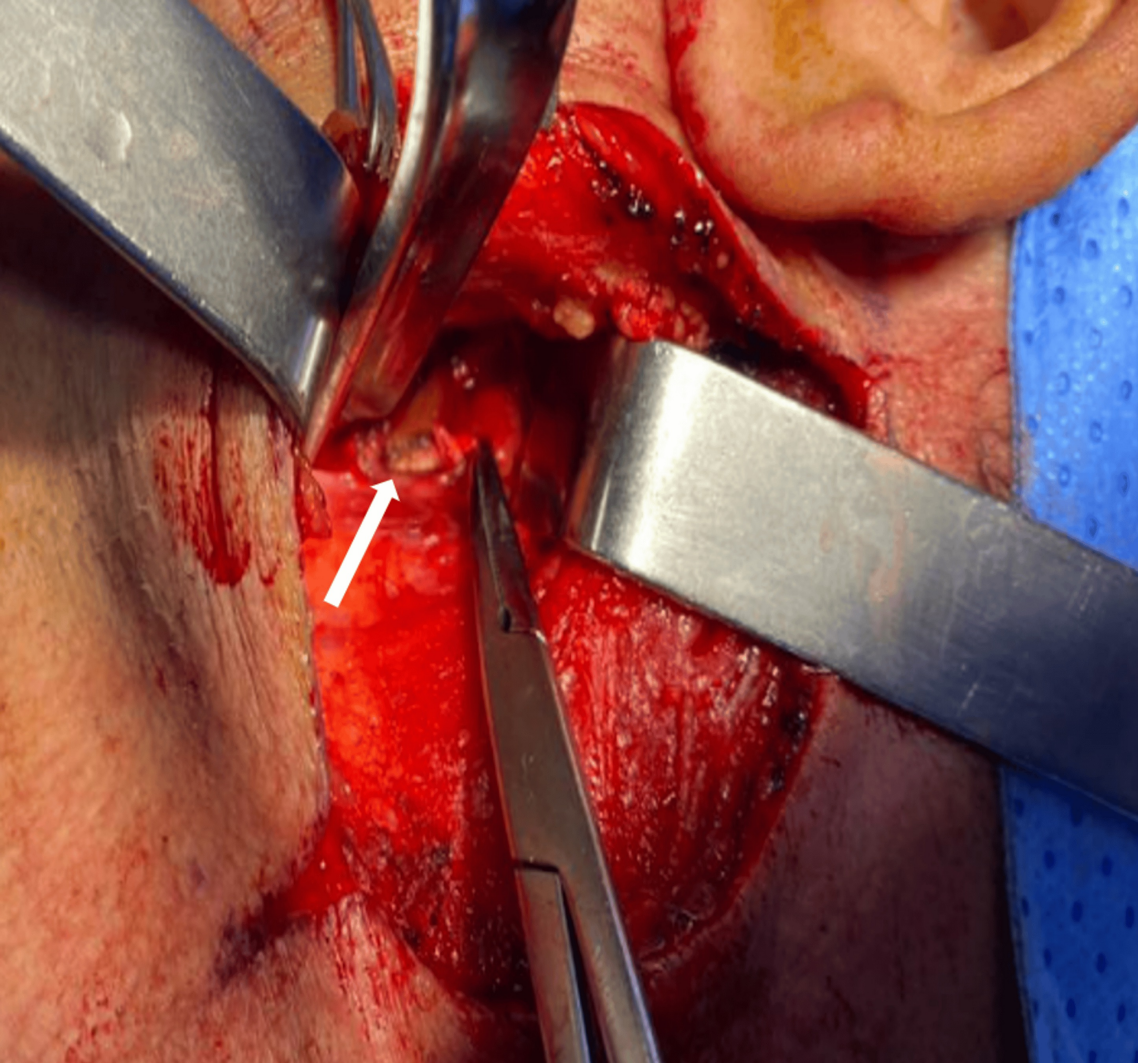
Case 6 intraoperative image of the styloid process (white arrow) during resection in a patient who experienced an ischemic stroke due to Eagle syndrome

The cardiac workup of the seven patients, including electrocardiogram, transthoracic echocardiography, and bubble test with transcranial Doppler, revealed no abnormalities. No patient underwent a hypercoagulability workup, and none had a history of recent COVID-19 infection. IV thrombolysis followed by mechanical thrombectomy was performed in one patient. Therapeutic anticoagulation was initiated immediately in four patients and 24 hours after IV thrombolysis and mechanical thrombectomy in the fifth patient, using low-molecular-weight heparin during the acute phase (mean duration: 6 days), followed by direct oral anticoagulants and physiotherapy. Anticoagulation was withheld in one patient due to the high risk of hemorrhagic transformation from a large ischemic lesion, and in another one (case 7), pending confirmation of the bony abnormality as the cause of the ischemic event, with no dissection or aneurysm and extrinsic compression confirmed by dynamic ultrasound; both were treated with aspirin. Styloidectomy was performed in three patients (Figure 5). In the patient with bilateral Eagle syndrome, surgery on the contralateral styloid process was performed a few months later. One patient underwent surgical resection of the greater horn of the hyoid bone. The postoperative course was uneventful. The mean interval between ischemic stroke and surgical treatment was nine months. Following surgery, curative anticoagulation was switched to aspirin (100 mg/day). Patients who have not yet undergone surgery remain on therapeutic anticoagulation. Discontinuation of aspirin after surgery is planned six months following surgical correction of the bony abnormality.

One patient, who had a poor prognosis at admission (NIHSS 12), died of septic shock secondary to a pulmonary infection. No patient experienced recurrent ischemic events during the entire study period. At six months of follow-up, the mean NIHSS score was 2 (range 0-5), and the mean mRS score was 2 (range 0-4). Table 1 presents a summary of clinical, imaging, and therapeutic data.

**Table 1:** Clinical, imaging, therapeutic features, and outcomes of patients in our series LMWH: low-molecular-weight heparin, DOAC: direct oral anticoagulant, M: male, F: female, ICA: internal carotid artery

Case	Age (years)	Sex	Delay of admission (hours)	Clinical presentation	Non-contrast CT scan	CTA	Bony stroke subtype	Acute phase treatment	Surgical treatment	Outcome
1	37	M	72	Right hemiplegia with aphasia; NIHSS = 17	Subacute left MCA stroke	Thrombus in the left MCA; ICA pseudoaneurysm in contact with an elongated styloid process (31 mm)	Vascular Eagle syndrome	DOAC (rivaroxaban 20 mg/day)	Not yet performed	Good NIHSS = 5, mRS = 3
2	66	M	12	Left hemiplegia; NIHSS = 9	Subacute right ACA stroke, right junctional MCA-ACA stroke	ICA dissection in contact with an elongated styloid process (32 mm)	Vascular Eagle syndrome	LMWH within 48 hours, followed by DOAC (rivaroxaban 20 mg/day)	Not yet performed	Good NIHSS = 1, mRS = 2
3	59	M	2	Left hemiplegia; NIHSS = 12	ASPECTS score 10	Thrombus in the right MCA; ICA dissection in contact with an elongated styloid process (75.8 mm)	Vascular Eagle syndrome	IV thrombolysis with mechanical thrombectomy. LMWH 24 hours later, for 4 days, followed by DOAC (rivaroxaban 20 mg/day)	Styloidectomy (performed 4 months later)	Good NIHSS = 3, mRS = 1
4	41	F	14	Left hemiplegia with homonymous lateral hemianopia; NIHSS = 14	Subacute right MCA Stroke	Floating thrombus in the ICA; compression of the carotid artery between the greater horn of the hyoid bone and the superior horn of the thyroid cartilage	Compression by hyoid bone and thyroid cartilage	LMWH for 10 days, followed by DOAC (rivaroxaban 20 mg/day)	Surgical removal of the greater horn of the hyoid bone (performed 18 months later)	Good NIHSS = 1, mRS = 1
5	49	F	2.5	Left hemiplegia with homonymous lateral hemianopia; NIHSS = 12	Subacute right MCA and ACA stroke	ICA dissection due to a conflict with the hyoid bone	Compression by the hyoid bone	Aspirin 100 mg/day	Not performed	Poor (death by septic shock)
6	57	M	28	Right hemiplegia with aphasia; NIHSS = 16	Subacute left MCA stroke Chronic right MCA ischemic lesion	Bilateral ICA dissection in contact with bilateral elongated styloid processes (42.3 mm on the left and 41.3 mm on the right)	Vascular Eagle syndrome	LMWH for 7 days	Left styloidectomy was performed 7 days later. Right styloidectomy was performed a few months later	Good NIHSS = 5, mRS = 4
7	45	M	4.5	Left hemiparesis; NIHSS = 6	ASPECTS score 10: chronic ischemic lesion in the ipsilateral MCA territory. MRI demonstrates sa ubacute right MCA stroke	Conflict between the right ICA and an elongated styloid process (37mm), without evidence of aneurysm or dissection. Velocity acceleration in the internal carotid artery during positional maneuvers, indicative of extrinsic arterial compression on dynamic ultrasound examination	Vascular Eagle syndrome	Aspirin 100 mg/day	Styloidectomy (performed 14 months later)	Good NIHSS = 0, mRS = 1

## Discussion

Structural and bony abnormalities are potential causes of ischemic strokes in both the anterior and posterior circulations, collectively referred to as bony stroke. There are different stroke mechanisms: direct arterial compression leading to embolism via endothelial damage or dissection; chronic arterial irritation resulting in pseudoaneurysm formation, which may serve as a source of emboli; or hemodynamic strokes caused by arterial stenosis. The conflict can be permanent or specific to certain head positions [2]. In this context, it is important to distinguish bony strokes from other mechanical causes of cervical artery dissection, including traumatic or spontaneous dissections unrelated to osseous structures.

From an anatomical perspective, four subtypes of bony stroke have been defined: vascular Eagle syndrome (elongated styloid process syndrome) is an abnormal contact between an elongated styloid process and the ICA, which can lead to ischemic stroke through arterial dissection, intimal injury with pseudoaneurysm formation, intramural hematoma, and luminal narrowing or occlusion [9]. The length of the styloid process is typically 25-30 mm, and a length of >30 mm is considered elongated [10]. The risk of cervical ICA dissection increases with an odds ratio of 1.08 for each additional millimeter of styloid process length, and there is also a correlation between the proximity of the styloid process to the ICA and the occurrence of dissection [11]. Thyroid cartilage variations are anatomical anomalies of the thyroid cartilage that can cause compression of the vertebral or ICA. Hyoid bone hypertrophy can compress the ICA, leading to ischemic stroke of embolic or hemodynamic origin. Vertebral or occipital anomalies, which can lead to vertebral artery stenosis or pseudoaneurysms, represent a potential source of emboli in the posterior circulation [2].

Bow hunter syndrome refers to dynamic compression of the vertebral artery, most commonly involving the V2 and V3 segments, by an osseous or fibrous structure during head rotation; however, other primary and secondary causes have also been described [2,4,12]. The atlantoaxial subtype is the most frequent. Clinical manifestations are variable, but the syndrome most often presents with vertigo, visual disturbances, and cerebellar stroke [12].

Bony strokes are an uncommon etiology of stroke in young adults, with a mean age of approximately 48-51 years [2,13,14]. In a five-year cohort, their incidence was 0.14% [2]. Eagle syndrome is slightly more frequent in men, as observed in our series [13-15]. Bony strokes tend to recur within the same arterial territory if not appropriately treated [2]. Therefore, evaluation for a bony abnormality should be included in the etiological work-up of recurrent ischemic strokes in the same territory and in the ICA dissection [3]. Appropriate treatment can not only reduce but also eliminate the risk of recurrence [2].

Diagnosis of bony stroke requires a combination of imaging modalities (CTA, MRA, digital subtraction angiography, and sonography of the cerebral vessels), including both static and dynamic assessments. The latter involves evaluating arterial anatomy in the neutral head position, during head rotation and reclination, and during swallowing to demonstrate arterial compression at specific positions [2,3,12,16]. Digital subtraction angiography remains the gold standard for diagnosing bow hunter syndrome; however, dynamic ultrasound and CTA are also useful diagnostic tools [4,17]. These modalities should identify the osseous structure responsible for the conflict and detect associated vascular complications, such as dissection, stenosis, or aneurysm [17]. All cervical vessels should be thoroughly assessed, as bony stroke can be bilateral in many cases. Eagle syndrome leading to bilateral dissection is observed in 26% of cases, as illustrated by case 6 in our series [2]. However, an etiological assessment should be performed to exclude all potential causes of stroke before attributing stroke to a bony conflict, as was done in our series [2].

The management of bony stroke requires a multidisciplinary approach that involves neurologists, neuroradiologists, interventional radiologists, anesthesiologists, and surgeons. Due to the rarity of case reports, there is currently no standardized treatment. Nonetheless, surgical removal of the compressing structure is well established to prevent recurrence, compared with antiplatelet therapy alone [16]. Treatment options are varied and include conservative management, endovascular stenting, vessel occlusion, especially for the vertebral artery, surgical bypass, and bone or cartilage removal [2]. When stenting is performed, it is important to note that persistence of the bony anomaly can lead to stent fracture and subsequent recurrence of ischemic stroke [2,18]. Conservative treatment is most often used as a temporary measure prior to surgical management. Surgical intervention has some limitations. For example, vertebral artery decompression for bow hunter syndrome does not restrict head movement. Still, it carries a risk of recurrence, whereas spinal fixation limits head movement and may adversely affect quality of life [12]. In the absence of global guidelines, treatment should be decided on a case-by-case basis within a multidisciplinary team, considering recurrence risk, quality of life, and surgical and anesthesia contraindications [12].

There is currently no available data in the literature regarding curative anticoagulation for bony stroke. In our series, anticoagulation was used successfully as a temporary or bridging strategy. It prevented stroke recurrence in cases 3, 4, and 6 prior to surgery, while cases 1 and 2 remain on DOAC therapy without recurrence. Given the small sample size, these observations should be interpreted with caution. Further studies are needed to compare anticoagulation as a conservative or bridging approach versus definitive surgical management.

In the series by Haertl et al., single-agent antiplatelet therapy was continued after successful endovascular treatment in two patients. It was discontinued 12 months after surgical management in one patient without stroke recurrence [2]. In a recent case report (2025), antiplatelet therapy was stopped nine months after surgery [16]. In our series, we elected to continue single-agent antiplatelet therapy for six months postoperatively, consistent with the European Stroke Organisation 2021 recommendations for carotid artery dissection, which suggest antithrombotic therapy for three to six months if restitution ad integrum of the arterial wall is achieved [19]. Further studies are required to establish specific recommendations for the management of bony stroke. Stroke recurrence was high in patients without surgical removal of the osseous anomaly, with a cumulative rate of 2.14 strokes per year, while no recurrence occurred in treated patients [2]. Although our series is small, it underscores the importance of considering bony anomalies as a potential cause of stroke in young patients, particularly in cases of recurrence.

This study has several limitations. The small sample size and the rarity of reported cases in the literature limit the generalizability of our findings. In addition, the retrospective design prevents establishing firm causal relationships. Finally, larger prospective studies are required to compare the efficacy of anticoagulant therapy alone versus surgical intervention in this patient population.

## Conclusions

Our series, along with multiple cases and series reported in the literature, highlights a rare yet treatable cause of stroke in young patients: bony stroke. They recur frequently within the same arterial territory if left untreated. They can affect both the anterior and posterior cerebral circulation. Diagnosis requires a combination of imaging modalities, including both static and dynamic evaluation of the arteries. All other potential etiologies should be excluded. No global treatment guidelines exist; however, management requires a multidisciplinary approach, with options ranging from conservative therapy to surgical removal of the bony structure or stenting, thereby allowing selection of the most appropriate intervention for each patient. Our study underscores the importance of examining bone structures in young stroke patients and highlights the potential role of curative anticoagulation as a bridging measure. Further studies are needed to establish standardized treatment strategies.
